# Cell Type-Specific Promoters of *Volvox carteri* for Molecular Cell Biology Studies

**DOI:** 10.3390/genes14071389

**Published:** 2023-07-01

**Authors:** Benjamin von der Heyde, Eva Laura von der Heyde, Armin Hallmann

**Affiliations:** Department of Cellular and Developmental Biology of Plants, University of Bielefeld, Universitätsstr. 25, 33615 Bielefeld, Germany

**Keywords:** *Chlamydomonas reinhardtii*, *Gaussia* luciferase, germ–soma division of labor, gonidia, light, *PCY1* promoter, *PFP* promoter, somatic cells, volvocine algae, *Volvox carteri*

## Abstract

The multicellular green alga *Volvox carteri* has emerged as a valuable model organism for investigating various aspects of multicellularity and cellular differentiation, photoreception and phototaxis, cell division, biogenesis of the extracellular matrix and morphogenetic movements. While a range of molecular tools and bioinformatics resources have been made available for exploring these topics, the establishment of cell type-specific promoters in *V. carteri* has not been achieved so far. Therefore, here, we conducted a thorough screening of transcriptome data from RNA sequencing analyses of *V. carteri* in order to identify potential cell type-specific promoters. Eventually, we chose two putative strong and cell type-specific promoters, with one exhibiting specific expression in reproductive cells (gonidia), the *PCY1* promoter, and the other in somatic cells, the *PFP* promoter. After cloning both promoter regions, they were introduced upstream of a luciferase reporter gene. By using particle bombardment, the DNA constructs were stably integrated into the genome of *V. carteri*. The results of the expression analyses, which were conducted at both the transcript and protein levels, demonstrated that the two promoters drive cell type-specific expression in their respective target cell types. Transformants with considerably diverse expression levels of the chimeric genes were identifiable. In conclusion, the screening and analysis of transcriptome data from RNA sequencing allowed for the identification of potential cell type-specific promoters in *V. carteri*. Reporter gene constructs demonstrated the actual usability of two promoters. The investigated *PCY1* and *PFP* promoters were proven to be potent molecular tools for genetic engineering in *V. carteri*.

## 1. Introduction

**Figure 1:**
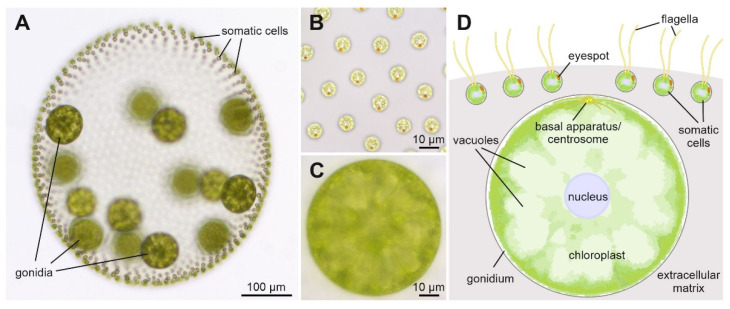
Phenotype and schematic cross-section of a *V. carteri* spheroid. (**A**) Wild-type phenotype of *V. carteri*, exemplified by an asexually grown female adult of strain Eve10. The alga consists of approximately 2000 small somatic cells and approximately 16 considerably larger gonidia, which are just before the beginning of embryogenesis. All cells are embedded in a transparent glycoprotein-rich extracellular matrix. The anterior pole of the spheroid is at the top of the image. (**B**) Close-up of the somatic cells. Top view from outside of the spheroid onto the flagellated pole of the somatic cells. All somatic cells are oriented so that their orange eyespots point to the posterior pole of the spheroid. (**C**) Close-up of a gonidium, with the focal plane bisecting the cell. (**D**) Schematic cross-section of a part of the *V. carteri* spheroid illustrating the arrangement of the cells and relevant subcellular structures. The small somatic cells, in contrast to the gonidia, have two flagella and an eyespot. To enable locomotion, they are localized at the surface of the spheroid. The large gonidia, which are located further inside the spheroid, are characterized by a large chloroplast and several large vacuoles arranged radially around the central nucleus.

Volvocine algae, a subgroup of the chlorophytes (green algae), span the full range of organismal complexity, from unicellular organisms, such as *Chlamydomonas reinhardtii*, to colonial organisms to multicellular organisms with a full germ–soma division of labor, such as *Volvox carteri*. *Volvox* is basically the simplest type of differentiated multicellular organism, as it has only two cell types: approximately 2000 small biflagellate terminally differentiated somatic cells, which form a monolayer at the surface of a sphere, and approximately 16 large flagella-less reproductive cells (gonidia), which lie just below the sheet of somatic cells (Figure 1). All cells are embedded in a transparent glycoprotein-rich extracellular matrix that holds the cells in place and can constitute up to 99% of the volume of the sphere. The alga is able to sense light stimuli and respond by changing its swimming behavior, an ability that is essential for optimizing photosynthesis. Most of the time, *Volvox* reproduces asexually, but when confronted with unpleasant conditions, such as a drying up pond, it switches to sexual reproduction and produces zygotes that are able to withstand harsh conditions [1,2].

For experimental studies of cell differentiation, *Volvox* offers the advantage that the different cell types can be mechanically separated from each other. The pure cell types can then be analyzed separately using molecular biological or biochemical methods for comparative studies [3,4,5].

In the last decades, the green microalga *V. carteri* has emerged as a useful model organism for studying aspects of multicellularity and cellular differentiation [2,6,7,8,9], photoreception and phototaxis [10,11,12,13,14], cell division [15,16], biogenesis of the extracellular matrix [17,18,19,20,21], and morphogenetic movements [1,22,23,24,25,26,27].

To answer a wide variety of questions in these subject areas, a broad range of molecular tools and bioinformatics resources have already been established for *Volvox* and also for its relatives.

The genomes of *V. carteri* [28] and the seven other volvocine algae, namely *C. reinhardtii* [29,30,31], *Tetrabaena socialis* [32], *Gonium pectorale* [33], *Astrephomene gubernaculifera* [34], *Pandorina morum* [35], *Yamagishiella unicocca* [35] and *Eudorina* sp. [35], are available at the plant genomics resource phytozome 13 [36] and/or GenBank [37,38,39]. These genomes offer a genome-wide comparison and analysis of differences between *V. carteri* and less complex volvocine species.

The transcriptome of *V. carteri* has been investigated with RNA-sequencing analyses focusing on protein-coding gene expression [4,5] and the micro-RNA repertoire [40]. There are also transcriptome-wide studies on (cell type-specific) alternative splicing [41,42].

For genetic-engineering experiments with *V. carteri*, stable nuclear transformation was established using the particle bombardment or biolistics technique [43]. Integration into the nuclear genome is mostly by illegitimate recombination, resulting in ectopic integration of introduced DNA, but homologous recombination is also feasible [44]. Different selectable marker genes for auxotrophic or antibiotic selection can be efficiently co-transformed with non-selectable genes of interest [43,45,46,47,48,49,50]. There are also several reporter genes for *V. carteri* that code for fluorescent [10,51,52], light-producing [53], and chromogenic reporter proteins [47]. For in vivo localization studies of key proteins, the corresponding genes are routinely fused to the reporter gene *yfp*, and the fluorescent fusion proteins are located in transformants, using confocal laser scanning microscopy [10,15,20,54]. Transposons of *V. carteri* can be used for transposon mutagenesis to randomly generate mutant phenotypes and then use the transposon sequence in these mutants to identify the insertion site [55,56,57]. Selective knockdown of target genes using RNA interference is also feasible [58]. Furthermore, endogenous genes can be edited or knocked out by CRISPR/Cas9 mutagenesis [59].

In a wide variety of genetic-engineering applications, the availability of well-defined endogenous promoters is crucial. Depending on the application; the use of strong, intermediate, or weak promoters; and whether it is constitutive or inducible, developmental-stage-specific or stage-enriched, or cell type- or tissue-specific promoters are required. Combinations of these are also needed, such as inducible weak or cell type-specific strong promoters. For some applications, promoters have already been identified and tested in *V. carteri*. In terms of inducibility, there are established promoters inducible by the *V. carteri* sex-inducer (pherophorin-S promoter) [60], nitrate (*nitA* promoter) [53], sulfur deprivation (arylsulfatase promoter) [47,61], and heat shock (*hsp70A* promoter) [49]. Strong constitutive promoters are available with the *rbcS3* promoter [49], the β-tubulin promoter [15,44,60], and the *Lhcbm1* promoter [10,15]. Another established promoter is the *isg* promoter, which is specific for the developmental stage of embryonic inversion [47]. To date, however, no cell type-specific promoters have been established for molecular cell biology studies in *V. carteri*. This is quite astonishing, as a major reason for selecting *Volvox* as a model system is that this simple multicellular alga shows a complete germ–soma division of labor, and even approximately 54% of all genes show cell type-specific expression [4]. It is then obvious to look for the molecular differences between the two cell types, for which cell type-specific promoters would be a helpful tool. By using such cell type-specific promoters, it is possible to perform cell type-specific labelling, knockdown, knockout, or overexpression of a gene of interest.

To make cell type-specific promoters usable in *V. carteri*, we screened transcriptome data from RNA-sequencing analyses and finally selected two supposed strong and cell type-specific promoters—one for gonidia-specific expression and one for specific expression in somatic cells. Both promoter regions were cloned and then placed in front of a reporter gene, the *G-Luc* gene, which encodes a luciferase. The corresponding DNA constructs were stably integrated into the genome of *V. carteri* transformants by particle bombardment. Expression analyses at both the transcript and protein levels showed that the two promoters indeed mediate cell type-specific expression in the respective target cell type, with expression strengths varying between different transformants.

## 2. Materials and Methods

### 2.1. MA-Plot (Bland–Altman Plot)

The analysis of cell type-specific expression was based on RNA-Seq data obtained from a previous whole-transcriptome analysis for *V. carteri* [4]. Mapping, data analysis, and bioinformatics are described there. The earlier study involved synchronized *Volvox* algae at the developmental stage just before the onset of embryogenesis, and the two cell types of *Volvox* were mechanically separated from each other and analyzed independently. The requirements for the determination of expression levels and cell type-specific expression were as previously described [4]. To analyze and visualize the expression data, we used the short-read mapping analysis platform ReadXplorer 2.2.3 [62], which also includes the R package DESeq [63,64,65]. DESeq was used for count data normalization and the calculation of mean values (baseMean) and fold differences in expression. Differential expression was tested using DEseq calculations [63] and Benjamini–Hochberg multiple testing adjustment [66], with a false discovery rate (FDR) of q = 0.1. An average baseMean expression value greater than 12.5 was sufficient for robust expression analysis (cutoff at 12.5).

### 2.2. Strains and Culture Conditions

*V. carteri* f. *nagariensis* strain Eve10 is a female wild-type strain from Japan that has been previously described [67,68,69]. In transformation experiments, the nitrate reductase-deficient (*nitA*^−^) strain TNit-1013 [10], a descendant of Eve10, was used as the recipient strain. Since the recipient strain cannot utilize nitrate as a nitrogen source, it was grown in standard *Volvox* medium [70] supplemented with 1 mM ammonium chloride (NH_4_Cl). Transformed *Volvox* algae that had the nitrate reductase gene complemented were grown in standard *Volvox* medium without ammonium chloride. All cultures were grown at 28 °C in a cycle of 8 h darkness, followed by 16 h of cool fluorescent white light [71] at an average of approximately 100 μmol photons m^−2^ s^−1^ photosynthetically active radiation (PAR). The *Volvox* algae were cultured in either glass tubes with caps allowing for gas exchange or in Fernbach flasks, which were aerated with approximately 50 cm^3^ sterile air/min.

### 2.3. PCR Amplification of DNA Fragments for Vector Construction

The extraction of genomic DNA of the wild-type *V. carteri* strain Eve10 was as previously described [72]. To amplify genomic DNA fragments containing promoter and terminator regions, PCR reactions were performed on a Mastercycler Gradient PCR thermal cycler (Eppendorf, Hamburg, Germany), using the genomic DNA of strain Eve10 as a template and specific oligonucleotide primers. Similarly, other components for vector construction were generated using plasmids as templates. Additional artificial restriction sites were previously added to the 5′ ends of the primers to facilitate the sequential connection of all DNA fragments during vector construction. The PCR conditions were as follows: initial denaturation at 98 °C for 2 min, followed by 35–40 cycles of 98 °C for 30 s, 55–60 °C for 30 s, and 72 °C for 30–60 s; the final elongation step was at 72 °C for 7 min. PCR products were analyzed by agarose gel electrophoresis and purified from the gel, using the GeneJET Gel Extraction Kit (Thermo Fisher Scientific, Wilmington, DE, USA) according to the manufacturer’s manual.

### 2.4. Construction of Vectors for Cell Type-Specific Expression of Luciferase

To construct the expression vector pPCY1-Luc, four fragments were required: the *PCY1* promoter region (Vocar.0015s0197), the *G*. *princeps* luciferase gene (*G-Luc*) [73], the *Lhcbm1* terminator region (Vocar.0001s0479), and the pBluescript II SK (-) vector (Agilent Technologies, Santa Clara, CA, USA) as a backbone. *PCY1*, *G-Luc*, and *Lhcbm1* fragments were generated by PCR, as described above. To amplify the coding sequence of *G-Luc*, a plasmid, pPsaD-GLuc, was used as a template [73]. The *G-Luc* on this plasmid was previously adapted to the nuclear codon usage of *C. reinhardtii* [73]. The three PCR fragments with artificial restriction sites at their ends were sequentially inserted into the vector backbone. The first inserted fragment (1.3 kb) contained the promoter region of the *PCY1* gene with the 5′UTR of the gene also included (artificial *Xho*I to artificial *Cla*I). The second fragment contained the 0.6 kb *G-Luc* coding sequence (artificial *Cla*I to artificial *Xba*I). The third fragment contained the 0.3 kb terminator region of the *V. carteri Lhcbm1* gene (artificial *Xba*I to artificial *Not*I).

To construct the expression vector pPFP-Luc, the same procedure as for pPCY1-Luc was followed, except that the promoter region and the 5′UTR, a fragment of 1.2 kb, in this case came from the *V. carteri PFP* gene (Vocar.0032s0153). In pPFP-Luc, the *G*. *princeps G-Luc* gene is under control of the *PFP* promoter region and has the *Lhcbm1* terminator region.

### 2.5. Stable Nuclear Transformation of V. carteri by Particle Bombardment

A logarithmically growing culture of *V. carteri* strain TNit-1013 was harvested via filtration. The stable nuclear transformation of strain TNit-1013 was carried out as previously described [43] by using a Biolistic PDS-1000/He (Bio-Rad, Hercules, CA, USA) particle gun [74]. Gold microprojectiles (1.0 µm in diameter, Bio-Rad, Hercules, CA, USA) were coated according to earlier protocols [75,76]. Algae were co-bombarded with the selectable marker vector pVcNR15 [77] and a non-selectable vector, either pPCY1-Luc or pPFP-Luc. The plasmid pVcNR15 carries the intact *V. carteri nitA* gene complementing the stable *nitA*-mutation of strain TNit-1013. The functional *nitA* version on plasmid pVcNR15 contains only the first intron of *nitA*; all others were deliberately omitted. This makes the plasmid much smaller than with all introns, and once it is genomically integrated, this shorter version of *nitA* can be easily distinguished from endogenous *nitA* by PCR. To select transformants, the nitrogen source in the *Volvox* medium was switched from ammonium to nitrate. For this purpose, the bombarded algae were distributed on Petri dishes (9 cm diameter) with standard medium containing nitrate as the sole nitrogen source. From the sixth day on after particle bombardment, algae cultures were screened for green and living transformants (*nitA*^+^) in a background of numerous bleaching, unaltered organisms (*nitA*^−^) by dark-field stereomicroscopy (MZ16A, Leica, Wetzlar, Germany). Identified transformants were transferred to fresh selective medium for further cultivation.

### 2.6. Genomic PCR

Genomic DNA of potential *V. carteri* transformants was isolated as described above. Genomic integration of *nitA* was investigated by PCR amplification of a *nitA* fragment, using genomic DNA as a template and the oligonucleotide primers 5′-TGCGAGAGGAGCTTGATGAGC and 5′-CCTCCGAGAGTCGGATCGC. Similarly, the genomic integration of *G-Luc* was investigated by use of the oligonucleotide primers 5′-GGATGTCCACGATGGCCTCG and 5′-ATCTAGATTACGTATCGTCGCCGCCG. PCR conditions were as follows: initial denaturation at 98 °C for 2 min, followed by 40 cycles of 95 °C for 30 s, 55 °C for 30 s, and 72 °C for 60 s; the final elongation step was at 72 °C for 7 min. Amplified PCR fragments were analyzed by electrophoresis, using 1.5 % agarose gels and a GeneRuler 100 bp DNA ladder (Thermo Fisher Scientific, Wilmington, DE, USA).

### 2.7. Fast Luciferase Screening Assay

For the large-scale phenotypic screening of transformants, 2 mL of a dense algal culture was concentrated on a 100 µm nylon screen and resuspended in 400 µL of assay buffer (0.1 M K2HPO4 pH 7.6, 0.5 M NaCl, 1 mM EDTA) [73]. The algae cells were then disrupted by 15 s of ultrasound sonication, using a Sonopuls HD2070 sonicator (Bandelin Electronic, Berlin, Germany) at 70% power, under cooling with ice. Based on the measured chlorophyll concentration of each sample, the samples were equalized for lysate concentration by adding assay buffer. In a black 96-well polystyrene plate (Thermo Fisher Scientific, Wilmington, DE, USA), 200 µL of each lysate was added to 50 µL of 10 µM coelenterazine (Fluka, Neu-Ulm, Germany) in assay buffer, using a multichannel pipette. Chemiluminescence was detected with a ChemiDoc MP chemiluminescence imager (Biorad, Hercules, CA, USA) and quantified using FIJI (ImageJ 1.51w) [78]. To document the uniformly adjusted lysate concentration of all samples based on their chlorophyll content, brightfield images were taken using a Nikon D200 camera.

### 2.8. Separation of the Cell Types of V. carteri

One liter of a *V. carteri* culture containing approximately 10,000 synchronously grown algae at the developmental stage shortly before onset of cell divisions was harvested on a 100 µm nylon screen. The concentrated algae were disrupted without lysing the cells, using a Dounce homogenizer with a tight-fitting pestle. The resulting suspension was transferred to the 100 µm nylon screen and washed with *Volvox* standard medium, allowing for the separation of soma sheets from gonidia: soma sheets are retained by the nylon screen while the gonidia pass through the screen. However, both fractions are not yet completely sorted then. The gonidia fraction from the flow-through was concentrated on a 10 µm nylon screen. To separate the gonidia from the remaining cell debris and singled somatic cells, the concentrated flow-through was transferred to a 50 mL Falcon tube that was filled up with *Volvox* standard medium and then centrifuged at 500× *g* for 2 min. The pelleted gonidia were then subjected to this washing process a second time. The resulting pellet was transferred to a 1.5 mL tube, 1 mL of medium was added, and the cell suspension was centrifuged at 500× *g* for 90 s. The supernatant liquid was then removed, and the pure gonidia in the pellet were brought to a final volume of 500 µL. The soma sheets that remained on the 100 µm nylon screen during the initial filtration also had to be cleared of individual cells of the other cell type. As some gonidia are tightly bound to the soma sheets, the soma sheet fraction was intensively treated with the Dounce homogenizer and transferred again to the 100 µm nylon screen and washed thoroughly. The retained soma sheets were transferred to a 1.5 mL tube, 1 mL of medium was added, and the cell suspension was centrifuged at 500× *g* for 5 min. The supernatant liquid was then removed, and the pure soma sheets in the pellet were brought to a final volume of 500 µL.

### 2.9. Isolation of Total RNA from Separated Cell Types

The homogenization and lysis of cells of soma sheet or gonidia suspensions were performed using a Precellys Evolution Homogenizer (Bertin Technologies, Montigny-le-Bretonneux, France). To this end, 250 µL fractions of each of the cell suspensions were transferred to 2 mL screw-cap tubes; ten 1 mm zirconia/silica beads (BioSpec Products, Bartlesville, OK, USA) and 1 mL phenol-based TRI reagent (Sigma-Aldrich, St. Louis, MO, USA) were added to each fraction; the samples were placed in the homogenizer; and cells were then lysed in 3 cycles of 20 s at 10,000 rpm, with a 10 s cooling period between cycles. For the extraction of total RNA, 300 μL of trichloromethane was added to the resulting homogenate. RNA precipitation and RNA purification were performed as previously described [41,75,76,79]. RNA quality and quantity were checked by agarose gel electrophoresis and UV photometry, using a Nanophotometer UV/Vis spectrophotometer (Implen, Munich, Germany).

### 2.10. Quantitative Real-Time RT-PCR

The SensiFAST SYBR No-Rox One-Step Kit (Bioline, Memphis, TN, USA) and a CFX96 Touch Real-Time PCR Detection System (Bio-Rad, Hercules, CA, USA) were used for real-time RNA quantification. All real-time RT-PCR experiments were carried out in three biological replicates with technical triplicates. For the amplification of a 119 bp fragment of the *G-Luc* cDNA, the specific primers were 5′-GGATGTCCACGATGGCCTCG and 5′-CCTGAGCCACATCAAGTGCAC; and for the amplification of a 118 bp fragment of the *tbpA* cDNA, the specific primers were 5′-GTAGTGGCTACTGTGAATCTGG and 5′-GCTCTCTAATACGCATAATGACG. The *tbpA* gene (Vocar.0016s0293) has generally been established as a reference gene for quantitative gene expression studies [80] and, in particular, has proven to be a reference gene for comparing the two cell types of *V. carteri* [81]. The temperature conditions in the CFX96 Touch Real-Time PCR Detection System were as follows: reverse transcription was at 45 °C for 20 min, followed by polymerase activation at 95 °C for 2 min and 40 cycles of DNA amplification at 95 °C for 7 s, 55 °C for 12 s, and 72 °C for 7 s. Melting curves were recorded to check for the amplification of a single product. Melting curves were generated by heating from 60 to 90 °C, with 1 °C increments per 10 s. The final products of all real-time RT-PCR reactions were visualized using agarose gel electrophoresis to verify amplification of products of correct size. The relative expression level was calculated using the 2^−ΔCt^ method [82,83].

### 2.11. Quantification of Luciferase Activity in Separated Cell Types

As a measure of *PCY1* and *PFP* promoter activity in *Volvox* transformants, luciferase activity was quantified separately for each cell type. For this purpose, algae cultures containing approximately 10 spheroids per mL were harvested, and the two cell types were mechanically separated from each other. A volume of 250 µL of the obtained soma sheet or gonidia suspension was mixed with 350 µL sample buffer (1.5 mM Tris-HCl pH 7.8, 1 mM EDTA) [73]. The cells were then disrupted by 15 s of sonication, using a Sonopuls HD2070 sonicator (Bandelin Electronic, Berlin, Germany) at 70% power, under cooling with ice. The resulting lysates were fractionated in 300 µL aliquots, frozen in liquid nitrogen, and stored at −70 °C for at least 30 min and at most 24 h before further processing. The lysates were thawed on ice in the dark and centrifuged at 20,000× *g* for 5 min at 4 °C. Supernatants were diluted 1:100 with sample buffer, which was pre-chilled to 4 °C, and then measured in technical triplicates. For each measurement, twenty microliters of each of the diluted supernatants was mixed with 125 μL of assay buffer (0.1 M K_2_HPO_4_ pH 7.6, 0.5 M NaCl, 1 mM EDTA), which was tempered to 20 °C, and preincubated for 15 min at 20 °C in the dark. For the luciferase activity assay, fifty microliters of 0.01 mM coelenterazine (Fluka, Neu-Ulm, Germany) was added to the preincubated samples, and the relative brightness was determined in a Sirius-L tube luminometer (Berthold, Bad Wildbad, Germany). The relative brightness was recorded for 10 s, and the luciferase activity was output in relative light units (rlu). The luciferase activity of each sample was related to its chlorophyll concentration, which is proportional to the original cell density.

### 2.12. Determination of the Chlorophyll Concentration

The chlorophyll concentration of lysates was determined as a measure of culture density or cell density and used to normalize the recorded relative brightness of the luciferase assay. For the determination of chlorophyll concentration, 200 μL thawed lysate was mixed with 800 μL of acetone and incubated for 45 min at 20 °C in the dark. After centrifugation at 16,000× *g* for 5 min, the absorption of the supernatant was measured at 647 nm and 663 nm in an UV/Vis spectrophotometer (Ultrospec 2100 Pro UV/Vis Spectrophotometer, GE Healthcare, Uppsala, Sweden). The chlorophyll content was determined as previously described [84].

## 3. Results

### 3.1. Identification of Suitable Genes with Strong Cell Type-Specific Expression

**Figure 2:**
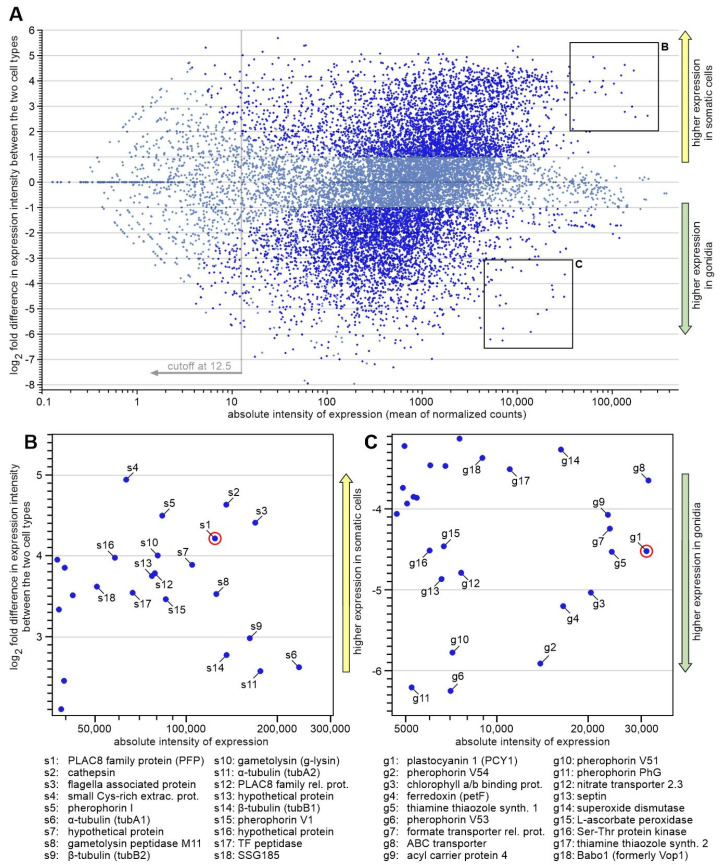
MA-plot of genome-wide gene expression data of both cell types. Each dot in this two-dimensional MA-plot represents both the absolute expression intensity of a given gene and the differences in expression of this gene between somatic cells and gonidia. More specifically, A-values (X-axis) represent the averages of absolute intensity of expression (mean of normalized counts) of a given gene in logarithmic scale, and M-values (Y-axis) represent the log_2_ fold difference in expression intensity of the same gene between the two cell types (somatic cells versus gonidia). Genes are classified as being expressed in a cell type-specific manner if the difference in expression between the two cell types is ≥2 and the significance value is ≤0.05, and they are shown with brilliant blue dots. Genes that do not meet these criteria are shown with light blue dots. The underlying RNA-Seq data originate from a previous whole transcriptome analysis [4]. (**A**) Full MA-plot (Bland–Altman plot) of the expression dataset. At total of 7691 genes (54%) show cell type-specific expression (brilliant blue dots). About half of them, more precisely 3728 genes (26%), show overexpression in somatic cells, and about half, more precisely 3963 genes (28%), show overexpression in gonidia. A total of 6556 genes (46%) do not show cell type-specific expression (light blue dots). Adapted from [4], with permission from BMC Biology, 2017. (**B**,**C**) Magnification of image sections highlighted in (**A**) showing genes with both very strong and cell type-specific overexpression in somatic cells and gonidia, respectively. Promoter regions of genes meeting these two criteria were considered candidates for cell type-specific overexpression of genes of interest in *V. carteri*. The names of the proteins encoded by the candidate genes are listed and further details are given in Supplementary Table S1. The two genes finally selected for expression analyses are circled in red in (**B**,**C**), respectively.

To identify suitable genes with promoters that could be used for the cell type-specific expression of any target gene in *V. carteri*, RNA sequencing data from a previous transcriptome analysis of separated cell types were used [4]. The two-dimensional MA-plot in Figure 2A is based on these expression data. Each dot shows both the absolute expression intensity of a given gene and the differences in expression of this gene between somatic cells and gonidia. The genes of particular interest for our candidate search are highlighted: genes that show both strong expression in general, i.e., lie as far to the right of the MA plot as possible, and show strong overexpression in either cell type, i.e., lie as close as possible to the upper or lower edge. As for overexpression in somatic cells, a group of candidate genes that fulfill these conditions is shown in Figure 2B. Similarly, a group of candidate genes with overexpression in gonidia is shown in Figure 2C.

From each of the two groups, 18 candidate genes were examined in more detail and compared with each other with respect to the following criteria: (i) overall expression level; (ii) extent of cell type-specific overexpression; (iii) quality of the gene models; (iv) matching of mapped RNA-seq reads with gene models; (v) predictability of the transcription start site; and (vi) availability of information on the possible function of the encoded protein. We obtained information on possible protein function by using the translated sequences of the *V. carteri* candidate genes to search with TBLASTN in the translated *C. reinhardtii* genome (v5.6) in Phytozome 13 [36] for the most similar protein sequence in *C. reinhardtii*. If there were hits with high sequence similarity (45 to 100%) and a function assignment in *C. reinhardtii*, we assumed an analogous function in *V. carteri*. The possible functions are given in Figure 2 and Supplementary Table S1, which also provides further details on the candidates. Based on the above criteria, we identified two genes/proteins as being most suitable: *PCY1* (Vocar.0015s0197) and *PFP* (Vocar.0032s0153). Further suitable genes/proteins can certainly be selected from both groups for later applications.

*PCY1* shows strong overexpression in gonidia and codes for plastocyanin, a small copper-containing protein that mediates electron transfer in photosynthesis. *PFP* shows strong overexpression in somatic cells and encodes a PLAC8 family protein, a small cysteine-rich plasma membrane protein that is putatively involved in cadmium resistance via heavy metal efflux. Both *PCY1* and *PFP* are within the 0.01% genes with the highest expression in the respective cell type.

### 3.2. Construction of Chimeric Genes for Promoter Analysis and Application

**Figure 3:**
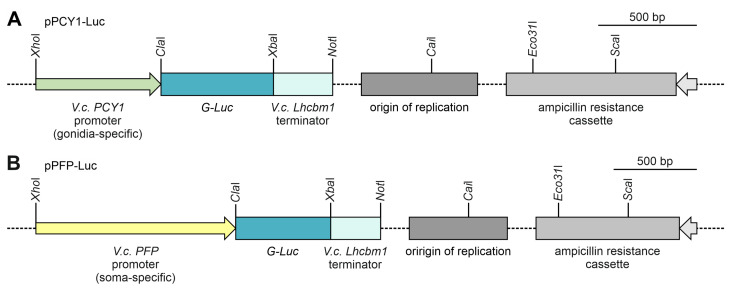
Schematic diagram of the transformation vectors pPCY1-Luc and pPFP-Luc. Both transformation vectors carry the coding sequence of the reporter gene *G-Luc* (570 bp), which is under the control of a gonidia- or soma-specific promoter, respectively. *G-Luc* encodes the luciferase of *G. princeps*. Both vectors also contain 292 bp of the terminator region of the *V. carteri Lhcbm1* gene. For propagation in *Escherichia coli*, the vectors have the backbone of pBluescript II SK (-), including an origin of replication of *E. coli* and an ampicillin-resistance cassette. The vector backbones are shown shortened (dashed lines). (**A**) In the transformation vector pPCY1-Luc, the reporter gene *G-Luc* is controlled by a 617 bp promoter region of the *V. carteri PCY1* gene (Vocar.0015s0197), which exhibits gonidia-specific expression. (**B**) In the transformation vector pPFP-Luc, the reporter gene *G-Luc* is controlled by a 1183 bp promoter region of the *V. carteri PFP* gene (Vocar.0032s0153), which exhibits soma-specific expression.

The promoters of *PCY1* and *PFP* were cloned from *V. carteri* genomic DNA and fused with a reporter gene, the *G-Luc* gene of the marine copepod *Gaussia princeps* coding for luciferase [73,85,86]. The version of the *G-Luc* gene used originates from the plasmid pPsaD-GLuc and was previously adapted to the nuclear codon usage of *C. reinhardtii* [73] but works just as well in *V. carteri* [53]. A major advantage of the *G-Luc* reporter gene is that a robust detection assay allows for the very sensitive detection of luciferase protein expression [53]. The artificial gene constructs also include a terminator region of the *lhcbm1* gene of *V. carteri* (Vocar.0001s0479) [10], which encodes a chlorophyll a/b binding protein. To simplify cloning and allow for the flexible replacement of parts of the construct, PCR was used to add artificial restriction sites to all DNA fragments at their ends. For the promoters, these restriction sites were *Xho*I and *Cla*I; for the *G-Luc* reporter gene, they were *Cla*I and *Xba*I; and for the terminator sequence, they were *Xba*I and *Not*I. The *Cla*I site is located immediately upstream of the start ATG of *G-Luc*, and the *Not*I site is immediately downstream of the stop codon. The DNA fragments were combined in a pBluescript II SK (-) backbone to obtain the vectors pPCY1-Luc and pPFP-Luc for transformation of *V. carteri* (Figure 3A,B). The sequences of the chimeric genes are given in Supplementary Figures S1 and S2.

### 3.3. Generation of Stable Transgenic Volvox Strains with Chimeric Genes

The recipient *V. carteri* strain TNit-1013 [10], which carries a stable mutation of the nitrate reductase gene (*nitA*), was co-transformed with the chimeric genes on vectors pPCY1-Luc and pPFP-Luc (Figure 3A,B), separately, and with a selectable marker plasmid (pVcNR15) carrying the functional *nitA* gene for complementation of the *nitA* mutation of TNit-1013 [77]. After 6 to 10 days of selection in a medium containing nitrate as the sole nitrogen source, algae cultures were screened for viable and healthy (green) cells among many dead (white) or dying (yellowish) cells. The number of transformants obtained was different for the two vectors pPCY1-Luc and pPFP-Luc (see below).

**Figure 4:**
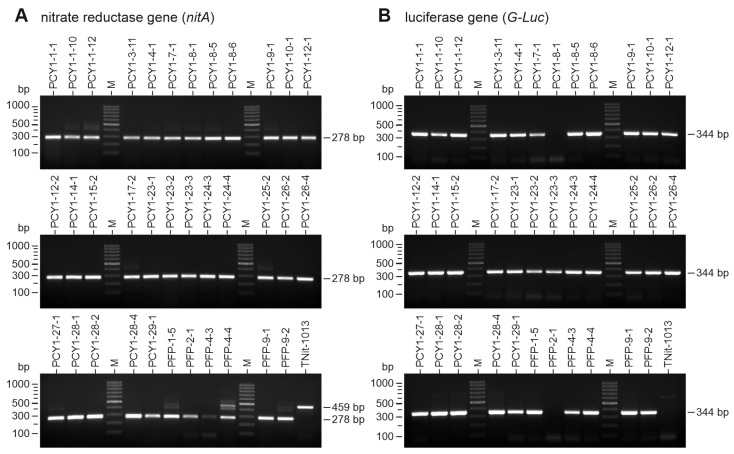
Proof of genomic integration of both *nitA* and *G-Luc* genes in transformants. Transformants capable of growing with nitrate as the sole source of nitrogen were analyzed for the presence of the *nitA* gene (**A**) and the *G-Luc* gene (**B**) in their genome by genomic PCR and agarose gel electrophoresis. Upon transformation, the *nitA* gene was located on the selectable marker vector, whereas the *G-Luc* was located on the second, co-transformed vector. The recipient strain TNit-1013 was used as a negative control. This PCR assay was performed more than 15 generations after the transformation of the strains to also test genetic stability. (**A**) The *nitA*-specific primers can bind to both the endogenous defective *nitA* gene and the functional *nitA* inserted during transformation, but different fragment sizes result. The defective *nitA* yields a 459 bp fragment, and the inserted *nitA* yields a 278 bp fragment. The reason for the size difference is that the fragment from the endogenous defective *nitA* contains the tenth *nitA* intron (181 bp), which is not present in the inserted functional version of *nitA*. If the inserted *nitA* is present in a transformant, the smaller 278 bp fragment prevails over the larger 459 bp fragment during PCR amplification. Since the recipient strain TNit-1013 has only the defective endogenous *nitA* gene, the larger, intron-containing 459 bp fragment is expected in this strain. (**B**) The *G-Luc*-specific primers can only bind to the reporter gene inserted during co-transformation. PCR amplification is expected to result in a 344 bp fragment if *G-Luc* is present. M-lanes refer to the molecular-weight marker, and the sizes of the marker bands are indicated on the left.

The integration of the vector-based genes into the genome of *V. carteri* transformants was investigated by genomic PCR. For this purpose, transformants first had to be able to grow for four weeks under selective pressure, i.e., in medium with nitrate as the sole nitrogen source, before their genomic DNA was isolated. Using *nitA*-specific primers and the isolated genomic DNA as a template, genomically integrated *nitA* fragments from the original transformation vector, pVcNR15, were amplified by PCR. From all 29 pPCY1-Luc transformants and all 6 pPFP-Luc transformants, a fragment of the expected size of 278 bp could be obtained (Figure 4A). It should be noted that the *nitA* primers not only allow for the amplification of the intronless *nitA* fragment of 278 bp that we were looking for but also of a larger (459 bp) fragment of the endogenous *nitA*, which is defective in the recipient strain. However, the smaller 278 bp fragment prevailed over the larger 459 bp fragment during PCR amplification (Figure 4A).

All transformants were also checked for genomic integration of the *G-Luc* reporter gene, using *G-Luc* specific primers. In 28 of 29 pPCY1-Luc transformants and 5 of 6 pPFP-Luc transformants, a fragment with the expected size of 344 bp was obtained (Figure 4B). In the case of two transformants (pPCY1-8-1 and PFP-2-1), there is no band, i.e., no co-transformation occurred with pPCY1-Luc or pPFP-Luc, and only *nitA* from vector pVcNR15 integrated into their genomes. As expected, there is also no band in the recipient strain TNit-1013 because it does not contain the *G-Luc* gene.

All experiments investigating genomic integration were performed more than 15 generations after the transformation experiments. Thus, the introduced chimeric genes studied were repeatedly passed from parent algae to their offspring, and this is possible only in the case of stable integration into the genome. The microscopic examination of all transformants revealed no abnormalities with respect to the phenotype compared to the recipient and wild-type strain (Supplementary Figure S3). Thus, the expression of luciferase under control of the *PFP* and *PCY1* promoters does not interfere with the normal development of the transgenic algae.

Based on all transformation experiments, we calculated a transformation efficiency of approximately 6 × 10^−5^ for pPCY1-Luc and 4 × 10^−5^ for pPFP-Luc. These values are similar to previously published transformation rates of approximately 2.5 × 10^−5^ [43], approximately 10^−6^ [46] and 0.6 to 1.2 × 10^−5^ [50].

The calculated co-transformation rate was 95% for pPCY1-Luc and 75% for pPFP-Luc. This is quite high compared to previous reports of 9 to 58% [50]; 40 to 80% [43]; 30% or 10 to 60% [46]; and 30 to 45 % [49]. Overall, the observed co-transformation rates appear to vary widely.

### 3.4. Fast Luciferase Screening Assay for General Detection of Transgene Expression

**Figure 5:**
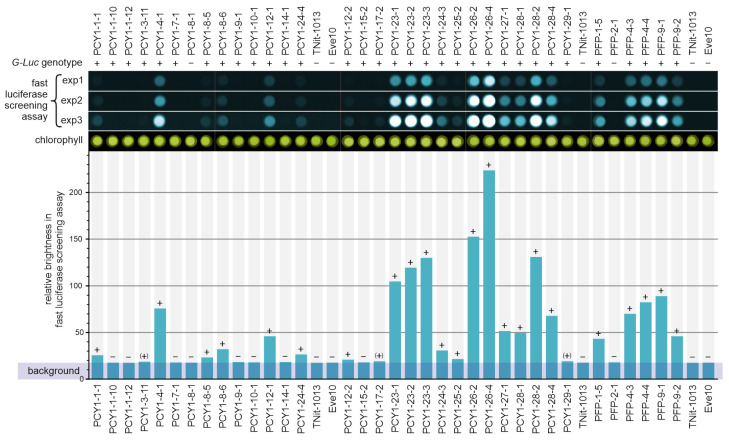
Fast luciferase screening assay of *V. carteri* transformants. Potential co-transformants that survived growth selection carried out with nitrate as the sole source of nitrogen were screened for expression of the *G-Luc* reporter gene under control of the *PCY1* promoter and the *PFP* promoter, separately, by measuring the luciferase activity. The chemiluminescence of cell lysates of the strains studied was detected immediately after the addition of the coelenterazine substrate, using a chemiluminescence imager. The chemiluminescence was integrated over three different exposure times (exp1 to exp3), allowing for a semiquantitative evaluation of the luciferase activity. Bright field photographs of the lysate samples document the amount of chlorophyll in each sample, reflecting the number of cells used, and show that all wells were loaded with comparable numbers of the cell lysates. The bar chart shows the relative brightness of all samples as a measure of luciferase activity and indicates whether luciferase activity above the background is not measurable (−) or can be measured (+) or whether there are only traces of activity ((+)). Both the wild-type strain Eve10 and the recipient strain TNit-1013 were used as negative controls because they cannot show luciferase activity and, thus, define the background level of the signal. For comparison of activity and genotype, the top row shows the result of genomic PCR to detect genome integration of *G-Luc* (Figure 4).

The demonstrated evidence of genomic integration of the transgenes does not guarantee their expression. Thus, the expression was also investigated using an enzyme assay: The *G-Luc* reporter gene utilized codes for a luciferase enzyme, which catalyzes the oxidation of coelenterazine while emitting light. For the enzyme assay, the enzyme only needs to be released by breaking the cells, but not purified. The cell lysates were generated by ultrasound sonication of complete spheroids of luciferase expressing *Volvox* transformants. Once the substrate is added to the cell lysates, a glow is visible even to the naked, dark-adapted eye in the darkroom if expression is strong enough. This method of detection is, of course, unsuitable as a measuring method. We therefore developed a semiquantitative assay for the fast screening of luciferase-expressing *Volvox* transformants. The number of processing steps, i.e., harvesting, cell lysis, incubation, and detection, was reduced to a minimum (see Section 2). In addition, the use of a chemiluminescence imager allows for the parallel measurement of many transformants, thus also enabling future large-scale applications. More than 15 generations after the transformation experiments, all transformants that had integrated both the *nitA* gene and the chimeric gene into their genomes were investigated for luciferase enzyme activity, using the fast luciferase screening assay (Figure 5).

As negative controls, a wild-type strain (Eve10), the recipient strain (TNit-1013), and two transformed strains without genomic integration of the co-transformed *G-Luc* vectors (PCY1-8-1 and PFP-2-1) were also tested. Considering the detected, albeit very low, light-emission in the tests with these four strains that do not have the *G-Luc* gene, we assume that a small fraction of the substrate is oxidized in this assay setup even in the absence of luciferase. Therefore, we defined a background level that is not attributable to luciferase activity. After subtracting this background, of the 28 pPCY1-Luc transformants with demonstrated genomic integration of the transgenes, 18 transformants showed clear luciferase activity due to *PCY1*-driven expression of *G-Luc* (Figure 5). Of the 5 pPFP-Luc transformants with proved genomic integration of the transgenes, all showed clear luciferase activity due to the *PFP*-driven expression of *G-Luc* (Figure 5). However, the measured luciferase activity varied greatly between the different transformants. This indicates that the *G-Luc* is expressed quite differently in different transformants, causing the enzyme amount to vary accordingly, a phenomenon generally observed in transgene expression. In the most highly expressing strain (PCY1-26 4), the light-emission, after subtraction of background, is more than 35-fold higher than in a strain with a clearly detectable but weak expression like that of PCY1-8-5. The wide range of expression strengths has the advantage here and in future applications that transformants with strong, medium, or weak expression can be selected depending on the objective. Transformants with medium–strong expression were selected for the following more detailed study.

### 3.5. Cell Type-Specific mRNA Expression of Transgenes Driven by PCY1 and PFP Promoters

**Figure 6:**
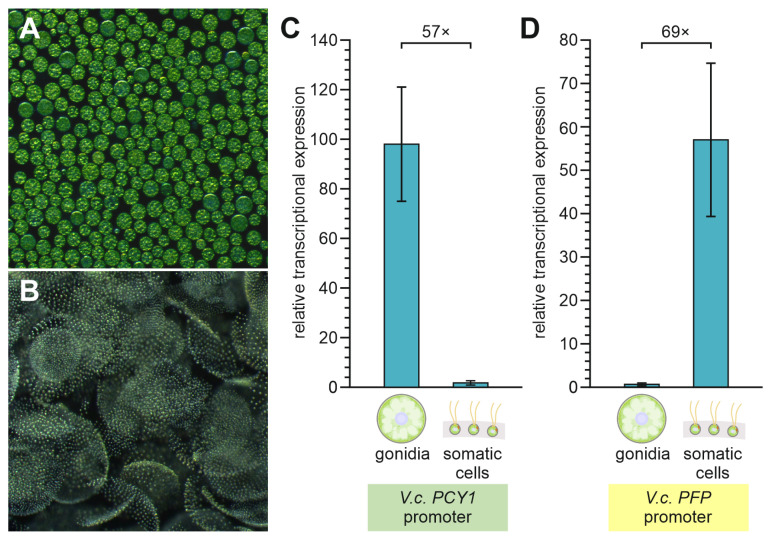
Quantification of *PCY1* or *PFP* promoter activity at the transcriptional level in gonidia and somatic cells of *V. carteri* transformants. Gonidia and somatic cells of transformants expressing the reporter gene *G-Luc* under control of the *PCY1* and *PFP* promoters, respectively, were mechanically separated just before the onset of cell division. Total RNA was extracted, and mRNA expression was determined by quantitative real-time RT-PCR. The relative transcriptional expression level reflects *PCY1*- or *PFP*-driven expression of *G-L*uc relative to the expression of the reference gene *tbpA* in the respective cell type. The calculation was performed using the 2^−ΔCt^ method. (**A**) Exemplary dark-field micrograph of separated gonidia. (**B**) Exemplary dark-field micrograph of separated somatic cell sheets. (**C**) Relative transcriptional expression of *G-Luc* under control of the *PCY1* promoter in transformant strain PCY1-26-2. The *PCY1* promoter causes overexpression of the reporter gene in gonidia, which is 57-fold higher than its expression in somatic cells. (**D**) Relative transcriptional expression of *G-Luc* under control of the *PFP* promoter in transformant strain PFP-1-5. The *PFP* promoter causes overexpression of the reporter gene in somatic cells, which is 69-fold higher than its expression in gonidia. The data represent three biological replicates with three technical replicates each. Error bars indicate standard deviations.

By analyzing mRNA expression separately for each cell type, we investigated whether the G-Luc transgenes driven by *PCY1* and *PFP* promoters show cell type-specific mRNA expression. For each of the two promoters used, a transformant with medium strong expression in the fast luciferase screening assay was selected as an example for this purpose, specifically PCY1-26-2 and PFP-1-5. In both strains, the two cell types were separated (Figure 6A,B) and total RNA was isolated from each of the two cell fractions. For measurement of cell type-specific mRNA expression of transgenes, quantitative real-time RT-PCR was performed using primers specifically for *G-Luc*, which is not present in the wild-type or recipient genome.

The endogenous reference gene used was *tbpA*, which has generally been established as a reference gene for quantitative gene expression studies [80] and, in particular, has proven to be especially useful as a reference gene for comparing the two cell types of *V. carteri* [81]. *PCY1*-driven expression in gonidia exceeds that of the reference gene by a factor of 98 (Figure 6C). Furthermore, *PCY1*-driven expression is 57 times higher in gonidia than in somatic cells (Figure 6C). For *PFP*-driven expression, the cell-type specificity and expression level are almost exactly the opposite of that for *PCY1*-driven expression. *PFP*-driven expression in somatic exceeds that of the reference gene by a factor of 57 (Figure 6D). Moreover, *PFP*-driven expression is 69 times higher in somatic cells than in gonidia (Figure 6D).

The expression of the *PCY1*- and *PFP*-driven transgenes in the transformants basically corresponds to the expression of the unmodified *PCY1* and *PFP* genes in the wild-type algae. For the transgenes in transformants, however, the differences in expression between the cell types are actually more pronounced than for the unmodified genes in the wild type. In the wild type, only a 23-fold-higher expression of *PCY1* was measured in gonidia compared to somatic cells, and only an 18.6-fold-higher expression of *PFP* in somatic cells compared to gonidia [4]. However, this may also be due to the different methods used, because expression of the transgenes in the transformants was performed here by quantitative real time RT-PCR, whereas expression of the unmodified *PCY1* and *PFP* genes in wild-type algae was performed by RNA-sequencing. It was observed earlier that RNA-sequencing-based transcriptome analysis tends to underestimate the cell-type specificity compared to real-time PCR-based single-gene expression measurements [4].

### 3.6. Quantification of PCY1- or PFP-Regulated Luciferase Activity Separately for Each Cell Type

Finally, after the investigation of transformants for cell type-specific mRNA expression of the *G-Luc* transgenes driven by *PCY1* and *PFP* promoters, cell-type specificity should be investigated at the protein expression level by measuring the luciferase enzyme activity. While the fast luciferase screening assay that was first used focused on the semi-quantitative rapid screening of a large number of transformants without cell-type separation, a more elaborate sensitive and quantitative measurement should now be performed on the separated cell types of selected transformants. Specifically, for each of the two promoters, three transformants with medium–strong expression in the rapid luciferase screening assay were chosen, namely for *PCY1*-transformants PCY1-23-2, PCY1-26-2, and PCY1-28-2 and for *PFP*-transformants PFP-1-5, PFP-4-4, and PFP-9-1.

**Figure 7:**
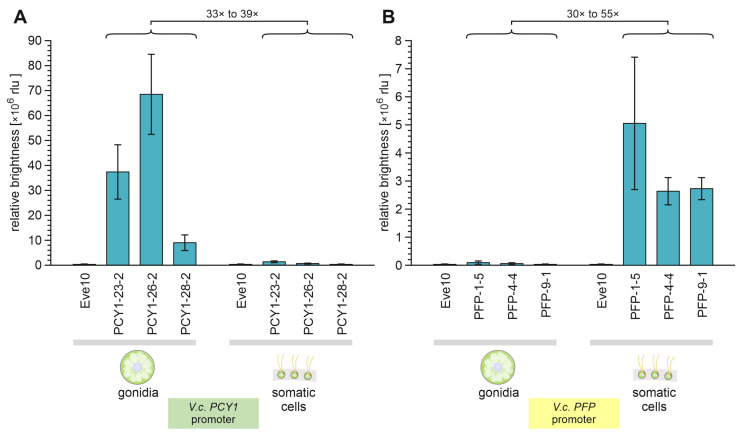
Quantification of *PCY1*- or *PFP*-regulated luciferase enzyme activity in gonidia and somatic cells of *V. carteri* transformants. The separated cell types of three independent transformant strains expressing the *G-Luc* reporter gene under control of (**A**) the *PCY1* promoter and (**B**) the *PFP* promoter, separately, were analyzed for luciferase activity. The bar chart shows the relative brightness of all samples in relative light units (rlu) as a measure of luciferase activity. The wild-type strain Eve10 was used as a negative control. To allow for a quantitative comparison of the luciferase activities, the determined enzyme activities of all samples were normalized with the respective chlorophyll content, which reflects the number of cells used. Columns represent the mean of three biological replicates with three technical replicates each. Error bars indicate standard deviations.

Transformants were grown under standard conditions, their cell types were separated, the cells were then lysed, and then the luciferase activity was quantitated in the supernatants after the addition of the coelenterazine substrate, using a Sirius-L tube luminometer (Berthold, Bad Wildbad, Germany). To allow for a quantitative comparison of luciferase enzyme activity, all measured luciferase activity values were normalized with the respective chlorophyll content of the samples, which is proportional to the original cell density. In contrast to the rapid luciferase screening assay above, this measurement yields a much better signal-to-background ratio. Therefore, the values of the negative controls defining the background level of substrate turnover that could not be attributed to luciferase activity appear to be particularly low in Figure 7.

The quantification of the luciferase expression driven by the *PCY1* promoter showed that the enzyme activity in the gonidia of transformants was 33-to-39-fold (average 35.8-fold) higher than in their somatic cells (Figure 7A). With the *PFP* promoter, it was the other way around: the quantification of the luciferase expression driven by the *PFP* promoter showed that activity in the somatic cells of transformants was 30-to-55-fold (average 35.5-fold) higher than in their gonidia (Figure 7B). Both *PCY1*-driven expression in somatic cells and *PFP*-driven expression in gonidia are extremely low, or in other words, each of the two promoters is almost completely off in one of the two cell types. Thus, the *PCY1* promoter allows for cell type-specific expression in gonidia, and the *PFP* promoter allows for cell type-specific expression in somatic cells.

The observed marked differences in the expression strengths between transformants that are produced in the same way are a known phenomenon that can also be exploited in expression experiments (see Discussion).

## 4. Discussion

In the course of this work, transcriptome data of *V. carteri* were screened for potential strong and cell type-specific promoters for *V. carteri*. Two of them were selected for experimental testing: one with potential strong gonidia-specific expression, the *PCY1* promoter, and one with potential strong soma-specific expression, the *PFP* promoter. These promoters were cloned, placed in front of a reporter gene, and integrated into the genome of *V. carteri*, using particle bombardment. Analyses of the transformants confirmed cell type-specific expression, with varying strengths among transformants. Experiments on the presence of the chimeric genes in the *V. carteri* genome took place more than 15 generations after the transformation experiments. Thus, the genomic integrations of the chimeric genes can be described as stable. In relation to the cell type-specific distribution of the *PCY1* and *PFP* mRNAs in wild-type, as identified through RNA-Seq analyses, both cloned promoters exhibit comparable cell type-specific expression of reporter genes in the transformants. Therefore, the cloned promoters function in the same way in front of the reporter genes as they do at their natural loci in front of their original genes. With these two promoters, the molecular toolbox of *Volvox* can now be expanded by two cell type-specific promoters, which are now available for further experiments.

Since the transfer of the promoter regions from the genome to the vectors did not change the properties of the promoters, it can be concluded that both the 617 bp promoter region of the *V. carteri PCY1* gene and the 1183 bp promoter region of the *V. carteri PFP* gene contain not only the core promoter but also all cis-acting regulatory elements required for cell-type specificity. In future studies, the transformation vectors pPCY1-Luc and pPFP-Luc will therefore allow us to shorten and mutate the promoter regions and then use the maintenance or disappearance of cell-type specificity in generated transformants to find out which DNA elements and motifs are actually required for cell type-specific expression. In addition to the identification of these DNA elements, the binding transcription factors could also be identified, e.g., with electrophoretic mobility shift assays.

We constructed the vectors pPCY1-Luc and pPFP-Luc in such a way that the reporter gene is flanked by two unique restriction sites (*Cla*I and *Xba*I). Therefore, the reporter gene can easily be exchanged against any other gene of interest.

In vectors pPCY1-Luc and pPFP-Luc, the promoter regions are also flanked by unique restriction sites (*Xho*I and *Cla*I). This design allows for the convenient substitution of these promoter regions with alternative cell type-specific or other promoter regions, enabling them to be tested in the same way.

The fast luciferase screening assay established in this work allows for the parallel measurement of a large number of transformants in future large-scale applications, using a chemiluminescence imager. For this rapid test, the cells of the transformants only need to be lysed; no further steps are necessary. However, this fast assay therefore only allows for a semi-quantitative evaluation of luciferase activity. For more accurate, sensitive individual measurements, a luciferase assay with a low signal-to-background ratio is also available. In this assay, the measured values are also normalized with the respective chlorophyll content of the samples, as this is proportional to the cell density.

As mentioned above, the expression strengths of the transgenes differed markedly between different transformants produced in the same way and with the same DNA constructs. This phenomenon is already known from previous expression studies in other *Volvox* transformants [53] or transformants of related green algae [75,76,79,87,88], or of higher plants [89] or animals [90]. The reasons for this can be different frequencies of insertion of the DNA constructs into the genomes of transformants [89], different position effects at the integration sites of the DNA constructs [91,92] since the integration happens randomly, and epigenetic gene silencing of transgenes [87,88,93]. However, the different expression strength of the transformants is not a disadvantage at all. Depending on the application, not only a particularly strong expression is desired but sometimes also a medium or weak expression. Due to the spectrum of transformants with different expression strengths, it becomes possible to select the transformant that possesses the most suitable expression strength. When opting for new promoters, however, care should be taken to ensure that the promoters express themselves as strongly as possible. It appears to be considerably easier to discover transformants with diminished expression compared to the wild-type gene, rather than those demonstrating enhanced expression.

The two cell type-specific promoters of *PCY1* and *PFP* that are now harnessed offer various capabilities. First, these promoters can be used to label different cell types with fluorescent proteins in different colors. This could help us study mutants more easily in germ–soma differentiations, such as *regA* mutants [55]. Furthermore, these promoters can be used to specifically manipulate individual cell types. This is achievable by cell type-specific RNAi knockdown or cell type-specific overexpression of a gene of interest exclusively in the desired cell type. It is also feasible to express cell type-specific key proteins, such as transcription factors in the wrong cell type to learn from the perturbations caused. With cell type-specific promoters, a rescuing transgene can also be expressed in only one cell type in an otherwise mutant background.

Profound genetic engineering of critical components usually turned out to be lethal [54,94] because components are frequently also involved in reproduction. Since in *Volvox* only gonidia are responsible for reproduction, it should be possible to specifically use the soma-specific promoter *PFP* to perform even severe manipulations of soma cells or their components that could even limit the viability of somatic cells. Especially soma-specific cell components such as flagella, eyespot apparatus, or the extracellular matrix could be studied with genetic-engineering experiments in this way. Even if the genes of interest are essential in both wild-type cell types, the manipulation in transformants can be restricted to the somatic cells only. As long as the gonidia remain capable of dividing and new reproductive cells are generated again, the organism should be able to be propagated under laboratory conditions. This could also allow overexpression or knockdown experiments, which cannot be performed in the closely related *Chlamydomonas* algae due to the lethality there, to be performed analogously in somatic cells of *Volvox*.

It can be assumed that there is even more potential for these cell type-specific promoters that is yet to be discovered.

## 5. Conclusions

Our aim was to harness cell type-specific promoters in *V. carteri*. Through screening and analyzing transcriptome data from RNA sequencing, we were able to make an initial selection. Eventually, we identified two promising and cell type-specific promoters—one for gonidia-specific expression, the *PCY1* promoter, and one for specific expression in somatic cells, the *PFP* promoter. By employing the *G-Luc* reporter gene, we constructed chimeric genes and conducted tests on both promoters. The results conclusively demonstrated that both promoters effectively mediate cell type-specific expression in the respective target cell types. The investigated *PCY1* and *PFP* promoters were proved to be potent molecular tools for genetic engineering in *V. carteri*.

## Data Availability

All data generated or analyzed during this study are included in this article and its supplementary information files. Plasmids are available upon request.

## Supplementary Materials

The following supporting information can be downloaded at https://www.mdpi.com/article/10.3390/genes14071389/s1: Supplementary Figure S1: Insert sequence of the vector pPCY1-Luc; Supplementary Figure S2: Insert sequence of the vector pPFP-Luc; Supplementary Figure S3: Phenotypes of wild-type, recipient, and transformant *V. carteri* strains; Supplementary Table S1: Genes and encoded proteins with both very strong and cell type-specific overexpression in somatic cells and gonidia, respectively.
