# Supplementary material for: Cell Type-Specific Promoters of Volvox carteri for Molecular Cell Biology Studies
Source: Genes (Basel). 2023 Jul 1;14(7):1389. doi: 10.3390/genes14071389 (PMC10379329; doi:10.3390/genes14071389)
Supplement: Supplementary file 1 [file genes-14-01389-s001.zip › Supplementary Figure_S3.pdf]

Supplementary Figure S3

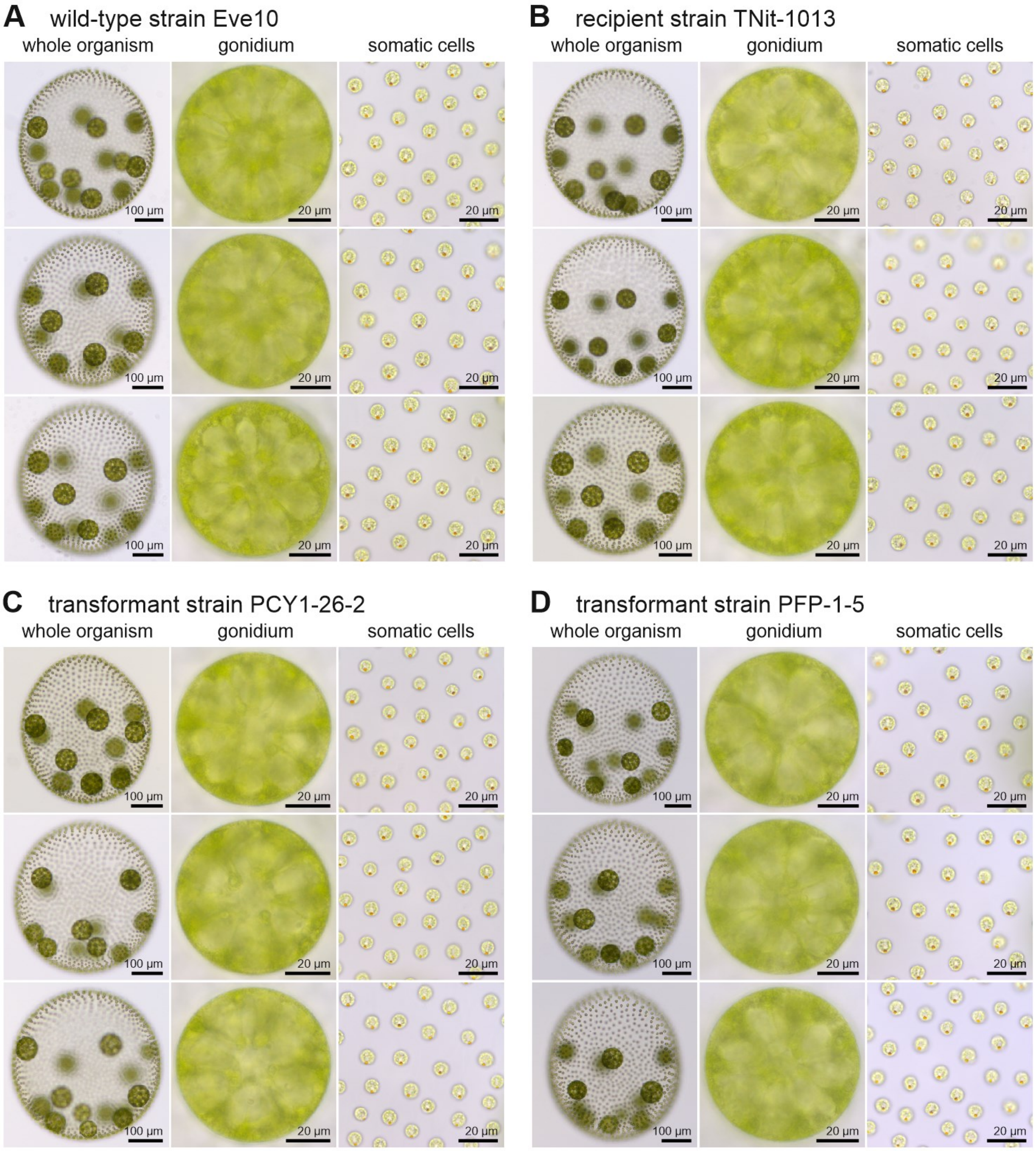

Phenotypes of wild-type, recipient and transformant *V. carteri* strains. For each strain, three example micrographs of the phenotype of the whole organism and enlarged views of a gonidium and some somatic cells are shown. Micrographs of whole organisms, gonidia and somatic cells were taken 4 h before onset of the dark phase of the 8 h dark/16 h light cycle, which corresponds to the stage just before onset of embryogenesis. **A** Wild-type strain Eve10. **B** Nitrate-reductase deficient recipient strain TNit-1013. **C** Transformant strain PCY1-26-2 expressing the reporter gene *G-Luc* under control of the *PCY1* promoter. **D** Transformant strain PFP-1-5 expressing the reporter gene *G-Luc* under control of the *PFP* promoter.
