# Supplementary material for: Cell Type-Specific Promoters of Volvox carteri for Molecular Cell Biology Studies
Source: Genes (Basel). 2023 Jul 1;14(7):1389. doi: 10.3390/genes14071389 (PMC10379329; doi:10.3390/genes14071389)
Supplement: Supplementary file 1 [file genes-14-01389-s001.zip › Supplementary Figure_S1.pdf]

Supplementary Figure S1

<u>CTCGAG</u>CGATCCGATTCCGAAGCATATCAGGGCATGCCGCAATTCAACCGGGATAAGGGGTTTATCATGTT
TCAATCCTTTGCGTCAAGGTTCGCAGGCTACCGTTGTCAGGTGAACGGTCATATCCACAGCAACGCCCCCC
GACGCGTACAGCCATGGCAAAATAAAAACATGTTGTGACACATCACAATATAATTGACCGGCTGGCACATG
CAAGGACAGCTCTTGACTGGATAGATATCCCTCGGCTGTCCGGACTGGTAATCCGCATGACGATCGGAGCA
GGCAACCTGGGAGGAATCTACGTTGTGTAATTAATTCAAAGTGAAAACTTTTTATATATCTGCAAGGTCAC
TACTTAAAGCACTTCAGTTGGAACACAGTATGATCATCTTGAATGTAACGAAGCCAACCTGCATACGTAAA
CAAATATATAAGTCTTCCAATCATCTCTATTTATCCATCCTCAATGTCTCTGGCCAATCTTATGCTGTCCA
TGTTCGTGCTACGGCTTCATGGGCCCTGTGGAATCTTATCATCCATCGTACTCGTTTGTTTCTATAAACAG
GCTGTCATTACCATCTATATTCACACCTGGTCACAGCGTATTAATCCATTCTCAT**<u>ATCGAT</u>**GAAGAG**ATG**G
TCAACGGCGTGAAGGTGCTGTTCGCCCTGATCTGCATCGCCGTGGCCGAGGCCAAGCCCACCGAGAACAAC
GAGGACTTCAACATCGTGGCCGTGGCCAGCAACTTCGCCACCACCGACCTGGACGCCGACCGCGGCAAGCT
GCCCGGCAAGAAGCTGCCCCTGGAGGTGCTGAAGGAGATGGAGGCCAACGCCCGCAAGGCCGGCTGCACCC
GCGGCTGCCTGATCTGCCTGAGCCACATCAAGTGCACCCCCAAGATGAAGAAGTTCATCCCCGGCCGCTGC
CACACCTACGAGGGCGACAAGGAGAGCGCCCAGGGCGGCATCGGCGAGGCCATCGTGGACATCCCCGAGAT
CCCCGGCTTCAAGGACCTGGAGCCCATGGAGCAGTTCATCGCCCAGGTGGACCTGTGCGTGGACTGCACCA
CCGGCTGCCTGAAGGGCCTGGCCAACGTGCAGTGCAGCGACCTGCTGAAGAAGTGGCTGCCCCAGCGCTGC
GCCACCTTCGCCAGCAAGATCCAGGGCCAGGTGGACAAGATCAAGGGCGCCGGCGGCGACGATACG**TAA**<u>**TC**</u>
<u>TAGA</u>TAAATTCCTGGTGGAGTCATTGTGTTGGAGCCATTGGAGCTCTGCAAGAGTGGCATTATCAAGAACG
TTCACGCGCGGAAGGGATATTTAGTGGCTGTCTGAGCGTAGAGTGATACCCACATCTTGGGTTTGTAACAT
AAATCTGATGTGATCCGATGTATCTTATCTATCTGTTCATTTGCTTGGACAATTTTCGGCATTATTAGGCA
GATGGACTGTGATAATGTAGAGCAGCCGAGAAATATGCACTGAAAGCAGGAAGCTAAACACGAAAACAATG
TATGCGGGCTCG**<u>GCGGCCGC</u>**

Xho I
PCY1 promoter region
Cla I
V.c. Kozak sequence
G-Luc
Xba I
Lhcbm1 terminator region
Not I

Insert sequence of the vector pPCY1-Luc. The vector pPCY1-Luc carries a 617 bp DNA fragment containing the *V. carteri PCY1*-promoter region, the 570 bp coding sequence of *Gaussia princeps* luciferase (*G-Luc*), and a 292 bp fragment containing the *V. carteri Lhcbm1* terminator region. The gene entries of *PCY1* and *Lhcbm1* in the current *Volvox carteri* genome version 2.1 in Phytozome 13 are Vocar.0015s0197 (*PCY1* gene) and Vocar.0001s0479 (*Lhcbm1* gene). The *PCY1* gene is on scaffold 15 at nucleotide positions 1482932 bp to 1484377 bp (forward). The *Lhcbm1* gene is on scaffold 1 at nucleotide positions 3740810 bp to 3742363 bp (forward). For the vector construction, pBluescript II SK (–) (Agilent Technologies, Santa Clara, CA, USA) was used as a backbone, because it includes an origin of replication of *E. coli* and an ampicillin resistance cassette. The sequence of pBluescript II SK (–) is as in GenBank (acc. no. X52330) but not shown here. The DNA fragments were inserted between the *Xho*I and *Not*I sites of the multiple cloning site of pBluescript II SK (–). The background colors indicate the following sequence features: green, *PCY1* promoter region; gray, *V. carteri* Kozak sequence; yellow, coding sequence of *G-Luc*; orange, *Lhcbm1* terminator region. Start and stop codons are highlighted (violet font). Relevant restriction sites are marked (bold, underlined).
