# Supplementary material for: Cell Type-Specific Promoters of Volvox carteri for Molecular Cell Biology Studies
Source: Genes (Basel). 2023 Jul 1;14(7):1389. doi: 10.3390/genes14071389 (PMC10379329; doi:10.3390/genes14071389)
Supplement: Supplementary file 1 [file genes-14-01389-s001.zip › Supplementary Figure_S2.pdf]

Supplementary Figure S2

**CTCGAG**CGAAGCGGTTCATCTGATGCGGAACCATCGGCACTCTGGAGCCCTACAGAGATTACTTAGGGTTT	*Xho* I
TAGGGTTTGACTCCCTGGACCGTATCCTGGAGCACATCAAGGGCCGACCCGCGGGTCAGTGGTCCATGGGC	
TCGCGGTTGTAGGGTTTTAGCTACATGGGTTGCGGTTTTAGGGTTTGCGGGAGCCCCACTGCTTGTTGGCA	
ATGGCCTTATTGGGCGAGGGGCGGTTCCGGGAGGGCCAGGCGGGCTAGTATGACGTGGCCCGCTCTGGAAC	
CCCCTCCTGCCCGTCAGGACTGACCGGCGTCTGACATTAAAATGCCTTGAAATAGGAGACTATACATAATT	
TACATGAAGACGCTCAAGATACAAAGAAACACAGTCGGCATATGTAAGTCGGCATGTGGCCCGGTGAGCCA	
AGGGCTGCCTTAGCGGCGTTACAGCATGGCCAAGACGCCAATGCGCGTCTTGGCTGAGGCTGCAGCGCGCA	
AGTGGAGACTGTGTTGCTAGACTAATTTGCTGGCGTCAAGTACGTCCAAAATGGTACTGGAAGTCAATGTA	
GGATATCGAATACAGCGAGAAATATAATATATGCGTATGGAAAGGAAGGATACTTTCTTCTGCTTATTCGT	*PFP* promoter region
CCCTTTCCAATCACAATATATCAACGCGCGCGTTGCGGCGAAATACAGACGACAGAAGTCCAGCGTGGATG	
TTGCTTCTACCTACTTAAGCTTGCTTTTTCCATAATTAGAGCTCAAAAACCTTTACAAAAACTGCAACCCA	
AAATCCGGACAAGCCAGCTTACCTATAACCAACTTTCAGTGTTGCATAACTACAATTTATACATCTTACAT	
TATACTGGACCTCTCGATTAACTGCGGCGGGCTTCCATGCCCTAGCCGTACCGGGAAGCGAAAGCTGCATT	
GACTTGTTACCAGGAAGAGCATCAAGTAAACAGGCACAAGTGGATAATGTTTATGATAAGGTTTAGACTAA	
CATTCTATTATCACCTGCTACGGCTCTCCAGTTGGGTCCAGTAGCCATACGGAACAGTTTGCGATACGGCC	
ATTGCGACAACTATTTCTTAGAATTGGCGTACATCCCTTGGTTCATAAGTCGCGAACTTTGATTGAGAGCG	
TACATAATAGTTTACCGTCACTACCTTTATAGTATTTCAGCGTCCCTATCCTT**ATCGAT**GAAGAG**ATG**GTC	*Cla* I
AACGGCGTGAAGGTGCTGTTCGCCCTGATCTGCATCGCCGTGGCCGAGGCCAAGCCCACCGAGAACAACGA	*V.c.* Kozak sequence
GGACTTCAACATCGTGGCCGTGGCCAGCAACTTCGCCACCACCGACCTGGACGCCGACCGCGGCAAGCTGC	
CCGGCAAGAAGCTGCCCCTGGAGGTGCTGAAGGAGATGGAGGCCAACGCCCGCAAGGCCGGCTGCACCCGC	
GGCTGCCTGATCTGCCTGAGCCACATCAAGTGCACCCCCAAGATGAAGAAGTTCATCCCCGGCCGCTGCCA	
CACCTACGAGGGCGACAAGGAGAGCGCCCAGGGCGGCATCGGCGAGGCCATCGTGGACATCCCCGAGATCC	*G-Luc*
CCGGCTTCAAGGACCTGGAGCCCATGGAGCAGTTCATCGCCCAGGTGGACCTGTGCGTGGACTGCACCACC	
GGCTGCCTGAAGGGCCTGGCCAACGTGCAGTGCAGCGACCTGCTGAAGAAGTGGCTGCCCCAGCGCTGCGC	
CACCTTCGCCAGCAAGATCCAGGGCCAGGTGGACAAGATCAAGGGCGCCGGCGGCGACGATACG**TAA****TCTA**	*Xba* I
**GA**TAAATTCCTGGTGGAGTCATTGTGTTGGAGCCATTGGAGCTCTGCAAGAGTGGCATTATCAAGAACGTT	
CACGCGCGGAAGGGATATTTAGTGGCTGTCTGAGCGTAGAGTGATACCCACATCTTGGGTTTGTAACATAA	*Lhcbm1* terminator region
ATCTGATGTGATCCGATGTATCTTATCTATCTGTTCATTTGCTTGGACAATTTTCGGCATTATTAGGCAGA	
TGGACTGTGATAATGTAGAGCAGCCGAGAAATATGCACTGAAAGCAGGAAGCTAAACACGAAAACAATGTA	
TGCGGGCTCG**GCGGCCGC**	*Not* I

Insert sequence of the vector pPFP-Luc. The vector pPFP-Luc carries an 1183 bp DNA fragment containing the *V. carteri PFP* promoter region, the 570 bp coding sequence of *Gaussia princeps* luciferase (*G-Luc*), and a 292 bp fragment containing the *V. carteri Lhcbm1* terminator region. The gene entries of *PFP* and *Lhcbm1* in the current *Volvox carteri* genome version 2.1 in Phytozome 13 are Vocar.0032s0153 (*PFP* gene) and Vocar.0001s0479 (*Lhcbm1* gene). The *PFP* gene is on scaffold 32 at nucleotide positions 1399706 bp to 1403755 bp (reverse). The *Lhcbm1* gene is on scaffold 1 at nucleotide positions 3740810 bp to 3742363 bp (forward). For the vector construction, pBluescript II SK (–) (Agilent Technologies, Santa Clara, CA, USA) was used as a backbone, because it includes an origin of replication of *E. coli* and an ampicillin resistance cassette. The sequence of pBluescript II SK (–) is as in GenBank (acc. no. X52330) but not shown here. The DNA fragments were inserted between the *Xho*I and *Not*I sites of the multiple cloning site of pBluescript II SK (–). The background colors indicate the following sequence features: green, *PFP* promoter region; gray, *V. carteri* Kozak sequence; yellow, coding sequence of *G-Luc*; orange, *Lhcbm1* terminator region. Start and stop codons are highlighted (violet font). Relevant restriction sites are marked (bold, underlined).
